# Assessing Risk in Focal Arboviral Infections: Are We Missing the Big or Little Picture?

**DOI:** 10.1371/journal.pone.0006954

**Published:** 2009-09-09

**Authors:** Andrew D. Haddow, Carl J. Jones, Agricola Odoi

**Affiliations:** 1 Department of Entomology & Plant Pathology, The University of Tennessee, Knoxville, Tennessee, United States of America; 2 Department of Comparative Medicine, The University of Tennessee, Knoxville, Tennessee, United States of America; Stanford University, United States of America

## Abstract

**Background:**

Focal arboviral infections affecting a subset of the overall population present an often overlooked set of challenges in the assessment and reporting of risk and the detection of spatial patterns. Our objective was to assess the variation in risk when using different at-risk populations and geographic scales for the calculation of incidence risk and the detection of geographic hot-spots of infection. We explored these variations using a pediatric arbovirus, La Crosse virus (LACV), as our model.

**Methods and Findings:**

Descriptive and cluster analyses were performed on probable and confirmed cases of LACV infections reported to the Tennessee Department of Health from 1997 to 2006, using three at-risk populations (the total population, the population 18 years and younger, and the population 15 years and younger) and at two geographic levels (county and census tract) to assess the variation in incidence risk and to investigate evidence of clustering using both global and local spatial statistics. We determined that the most appropriate at-risk population to calculate incidence risk and to assess the evidence of clustering was the population 15 years and younger. Based on our findings, the most appropriate geographical level to conduct spatial analyses and report incidence risk is the census tract level. The incidence risk in the population 15 years and younger at the county level ranged from 0 to 226.5 per 100,000 persons (median 41.5) in those counties reporting cases (n = 14) and at the census tract level it ranged from 50.9 to 673.9 per 100,000 persons (median 126.7) in those census tracts reporting cases (n = 51). To our knowledge, this is the highest reported incidence risk for this population at the county level for Tennessee and at the census tract level nationally.

**Conclusion:**

The results of this study indicate the possibility of missing disease clusters resulting from performing incidence risk investigations of focal diseases using inappropriate at-risk populations and/or at large geographic scales. Improved disease surveillance and health planning will result through the use of well defined at-risk populations and the use of appropriate geographic scales for the analysis and reporting of diseases. The finding of a high incidence risk of LACV infections in eastern Tennessee demonstrates that the vast majority of these infections continue to be under-diagnosed and/or underreported in this region. Persistent prevention and surveillance efforts will be required to reduce exposure to infectious vectors and to detect new cases of infection in this region. Application of this study's observations in future investigations will enhance the quantification of incidence risk and the identification of high-risk groups within the population.

## Introduction

The first step in the control and prevention of pathogen transmission requires the identification of the population at-risk. Early work involved the use of purely observational data to identify and prevent disease outbreaks, such as John Snow's calculation of the risk of death by water supply, leading to the identification of “contaminated” water supplies and efforts by the City of London to prevent the drinking of water from those sources [1]. Although today we have more advanced technologies at our disposal, the underlying principles of determining disease occurrence remain roughly the same. We use the incidence of disease to determine populations at-risk and cluster analyses to identify those areas at the highest risk for infection in an effort to guide strategies to interrupt and/or prevent transmission.

In this study, we explored the variability of incidence risk and investigated evidence of spatial clustering, using a focal arbovirus, La Crosse virus (LACV) as our model. LACV is a member of the genus *Orthobunyavirus*, family Bunyaviridae. It is the causative agent of LACV infections, and is one of the most common causes of pediatric arboviral encephalitis in the United States [2], [3]; the majority of cases are reported in children 15 years and younger [4], [5], [6], [7], [8]. Maintenance and transmission of the virus typically occurs in or near focal wooded areas where the primary vector, the eastern tree-hole mosquito, *Ae. triseriatus* [9], [10], [11], [12], and the primary amplification hosts the eastern chipmunk (*Tamias striatus*), the gray squirrel (*Sciurus carolinensis*), and the fox squirrel (*Sciurus niger*), are in close contact [2], [13], [14]. Transmission occurs when persons enter these focal areas and are bitten by infective mosquitoes.

LACV infections can be asymptomatic or symptomatic presenting as LACV fever, LACV meningitis, LACV encephalitis, or LACV meningoencephalitis [8]. Severe LACV infections can result in a variety of sequelae, including seizures, behavioral changes, learning disabilities, and cognitive deficits [15], [16], [17]. An early study in Wisconsin found that there were twice as many patients permanently institutionalized for mental disorders with LACV antibodies than in the general population [18]. Furthermore, when those patients with physiological cerebral defects were excluded from the analysis, the number of institutionalized patients with LACV antibodies increased to three times as many when compared to the general population. More recently, a follow-up study of pediatric patients who suffered from severe LACV infections in West Virginia found that 35.6% had full-scale IQ scores ≤79 post infection [15]. Patients with LACV infections may also experience a loss of social interactive skills, resulting in isolation from their peers and leading to difficulties in school and home environments [4]. Moreover, treatment during the course of a severe infection and post-infection can result in a high economic burden to the families of the patients [19], [20]. A recent study in North Carolina measured both the economic and social impacts of severe LACV infections and found that the projected life-long costs resulting from permanent neurological sequelae ranged from $48,775 to $3,090,798, and the loss of 12.90 to 72.37 disability adjusted life years (DALYs) [19].

Although LACV has traditionally been associated with the upper-Midwestern United States [4] it has been reported in other regions (i.e. Appalachia) and recently as an emerging disease in eastern Tennessee [21], [22], [23]. Nationally, there have been an average of 79 reported cases per year since 1964 [8]. However, the true incidence of LACV infections remains unknown as the disease is under-diagnosed and underreported [21], making detection and intervention by public health officials problematic.

From 1964 to 1996, there were 2370 total cases of LACV infections reported to the Centers for Disease Control and Prevention (CDC), with Tennessee reporting nine cases or 0.4% of the total number of cases during the time period. In 1997, 10 cases were detected in eastern Tennessee [21], during which time active surveillance for the virus was initiated by both the University of Tennessee and the Tennessee Department of Health. During the 10 year time period following increased surveillance efforts, 1997 to 2006, Tennessee reported 118 cases out of the 1069 cases reported to the CDC, accounting for 11.0% of all nationally reported cases, marking a substantial increase in the number of reported cases for Tennessee from the previous 32-year time period.

Focal diseases affecting a subset of the overall population present an often overlooked set of challenges in the calculation and reporting of incidence risk, and the detection of spatial patterns. We explored these unique challenges by examining cases of a focal pediatric arbovirus, LACV, in eastern Tennessee to determine the most appropriate at-risk population and geographic level to use in the investigation and reporting of disease risk.

## Methods

### Study area

**Figure 1:**
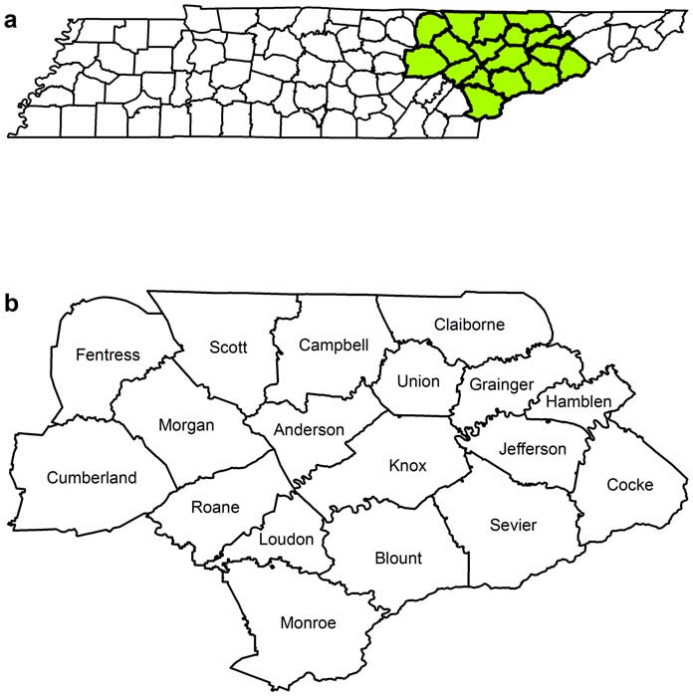
Maps of the study area. Map of Tennessee (a) showing the study area and (b) the distribution and names of the counties within the study area.

This study was conducted in eastern Tennessee (Figure 1). This area was chosen because it is endemic for LACV [21], [23], and the health department responsible for this region was specifically interested in the geographic epidemiology of the disease to inform their program planning. The study area was comprised of 18 counties: Anderson, Blount, Campbell, Claiborne, Cocke, Cumberland, Fentress, Grainger, Hamblen, Jefferson, Knox, Monroe, Morgan, Loudon, Roane, Sevier, Scott and Union (Figure 1). This area had a total population of approximately 1.1 million persons, of which approximately 0.25 million were children 18 years and younger, and approximately 0.2 million were children 15 years and younger. The median family income in the study area was approximately $37,000 a year.

### Data sources

#### La Crosse virus infection case data

Case data for probable and confirmed cases of LACV infections from 1997 to 2006 were provided by the East Tennessee Regional Health Department, upon written release by patients infected with LACV. Confirmed cases of LACV infection met both the clinical and laboratory requirements set by the CDC's case definition for neuroinvasive domestic arboviral diseases [8], [24], [25]. Cases that met the clinical definition and the initial antibody screening that detected virus specific antibodies, were classified as probable cases. Case data included information on the illness onset date, age, and the residential address. To protect patient confidentiality, personal identifying information was deleted before the database was released to investigators. This research was deemed exempt from review and certification by the University of Tennessee's Institutional Review Board following review by the Department of Entomology and Plant Pathology's Departmental Review Committee under the University of Tennessee's guidelines for research involving human subjects. Residential addresses were available for 15 probable and 76 confirmed cases of LACV infections reported during this time period. Probable and confirmed cases (n = 91) were combined for all analyses.

#### Population and geographic data

Population data was obtained from the 2000 United States Census [26] and was used as the denominator in the computation of incidence risk. The majority of LACV infections are pediatric [4], [5], [6], [7], [8]; therefore it is not appropriate to use the total population as the denominator when calculating risk. Hence, the use of the population 18 years and younger and the population 15 years and younger are the most appropriate populations for calculating incidence risk. To determine the existence of variation in incidence risk according to age, the total population, the population 18 years and younger, and the population 15 years and younger (three population groups) were used to compute incidence risk. To further assess if the observed spatial patterns were dependent on the geographic spatial level of analysis the incidence risk was calculated at both the county and the census tract spatial levels for all three population groups. Counties in the study area had an average population of 62,000 persons and were generally heterogeneous in relation to the population's socioeconomic and demographic characteristics, which are known determinants of health. In contrast, census tracts are subdivisions of a county that are typically homogenous in relation to socioeconomic and demographic factors. They contain an average of 4,000 persons, though populations can range from 1,500 to 8,000 persons [27]. Topologically Integrated Geographic Encoding and Referencing (TIGER) files containing cartographic boundary files were downloaded from the United States Census [28], and were used to display spatial patterns of disease risk.

### Statistical and geographic analyses

To investigate the spatial patterns of LACV infections incidence risk was calculated at both the county and the census tract levels, and spatial analyses were performed on 91 cases (100%) of all ages, 86 cases (94.5%) that were 18 years and younger, and 84 cases (92.3%) that were 15 years and younger. The incidence risk was calculated for all three population groups for all counties in the study area (n = 18) and for those counties reporting cases of LACV infections (n = 14). Incidence risk was also calculated for all three population groups for all census tracts in the study area (n = 230), for those census tracts reporting cases in the total population (n = 55), for those census tracts reporting cases in the population 18 years and younger (n = 52), and for those census tracts reporting cases in the population 15 years and younger (n = 51). Incidence risk was expressed as the number of cases per 100,000 persons. Descriptive analyses and the calculation of incidence risk were performed using STATA 10.0 [29].

To adjust for the high variances resulting from the small number of cases reported in some census tracts, we smoothed the incidence risk at the census tract level using spatial empirical Bayesian (SEB) smoothing [8], [30], [31], [32], [33] and specified inverse distance spatial weights to define the neighborhoods. This method allowed for better visualization of spatial patterns compared to the maps of unsmoothed incidence risk at the census tract level. Evidence of spatial clustering was assessed using the global Moran's I [34] and the Local Indicators of Spatial Association (LISA) [35], also using inverse distance spatial weights. Statistical significance of the global Moran's I and LISA statistics were tested using 9999 permutations. SEB smoothing and the computation of the Moran statistics were performed using GeoDa Version 0.95i [36]. Cartographic displays were made using ArcView GIS 9.2 [37]. Jenk's optimization classification scheme was used for the production of choropleth maps.

## Results

### Distribution of LACV infection incidence risk by at-risk population and geographic level

The incidence risk of LACV infection varied by the population at-risk and by the geographic level of analysis (Table 1). Higher LACV incidence risks were observed at the census tract level and in the younger populations (both the 18 and 15 years and younger age groups). In the population 18 years and younger, the county level LACV infection incidence risk in those counties reporting cases ranged from 3.9 to 188.4 per 100,000 persons (median 34.9), and in the census tracts reporting cases the incidence risk ranged from 44.3 to 547.1 per 100,000 persons (median 114.6) (Table 1). LACV infection incidence risk was the highest in the population 15 years and younger. In this population, the county level incidence risk in those counties reporting cases ranged from 4.7 to 226.4 per 100,000 persons (median 41.5), and for those census tracts reporting cases the incidence risk ranged from 50.9 to 673.9 per 100,000 persons (median 126.7).

**Table 1:** Comparisons of the Incidence Risk of La Crosse Virus Infections for At-Risk Population Groups at the County and Census Tract Levels in eastern Tennessee, 1997–2006.

	Incidence Risk per 100,000 persons					
	County Level			Census Tract Level		
Population Groups	n*	Median	Range	n*	Median	Range
Total population (All areas)	18	7.6	0.0–46.9	230	0.0	0.0–133.3
Total population (Only areas reporting cases)	14	8.8	0.9–46.9	55	27.7	11.9–133.3
Population 18 years and younger (All areas)	18	26.3	0.0–188.4	230	0.0	0.0–547.1
Population 18 years and younger (Only areas reporting cases)	14	35.0	3.9–188.4	52	114.6	44.3–547.1
Population 15 years and younger (All areas)	18	31.4	0.0–226.4	230	0.0	0.0–673.9
Population 15 years and younger (Only areas reporting cases)	14	41.5	4.67–226.4	51	126.7	50.9–673.9

* Number of counties or census tracts used for each specific analysis.

### Distribution of the geographic patterns of LACV infections

**Figure 2:**
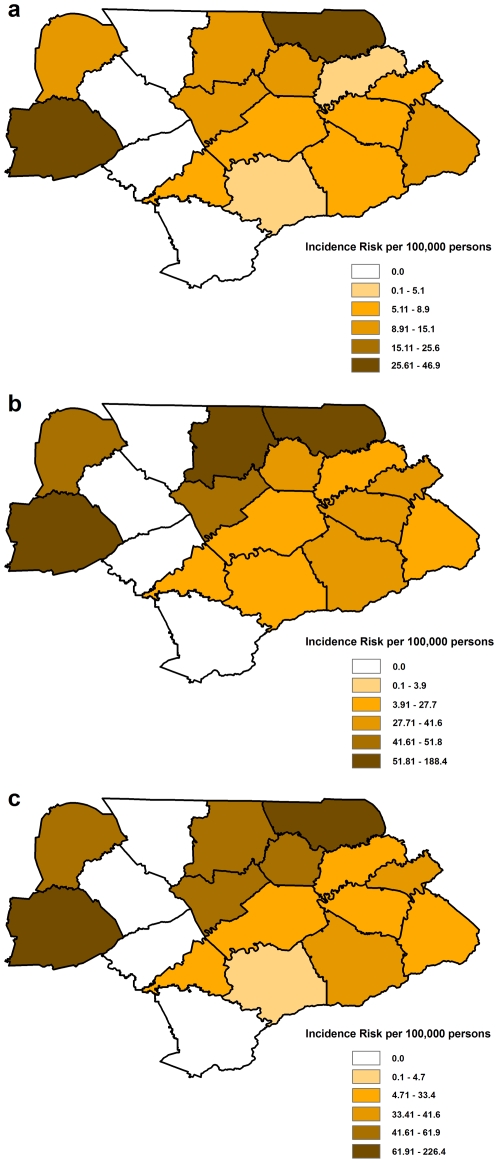
The unsmoothed incidence risk at the county level. These maps represent the distribution of unsmoothed risk of La Crosse virus infections at the county level for eastern Tennessee using three different population groups to calculate incidence risk: a) the total population, b) the population 18 years and younger, and c) the population 15 years and younger.

**Figure 3:**
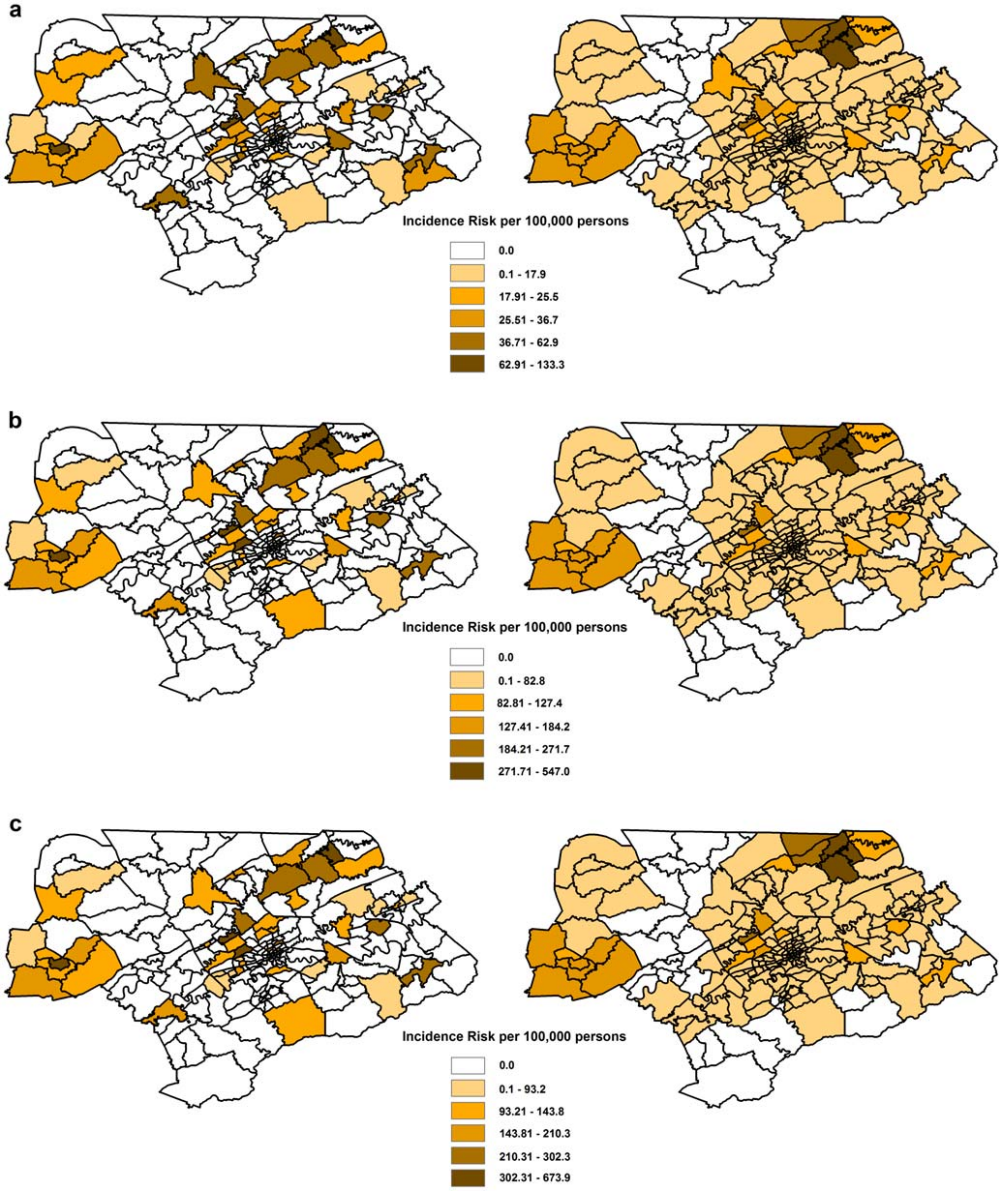
The unsmoothed and smoothed incidence risk at the census tract level. The maps in the left hand column represent the distribution of unsmoothed risk of La Crosse virus infections at the census tract level for eastern Tennessee. The maps in the right hand column represent the distribution of spatial empirical Bayesian (SEB) smoothed risk for La Crosse virus infections in the eastern Tennessee at the census tract level. Incidence risk was calculated using three different population groups: a) the total population, b) the population 18 years and younger, and c) the population 15 years and younger.

Geographically, the highest incidence risk was observed in the western and northeastern counties (Figures 2 and 3). Interestingly, the assessment of the spatial patterns at the census tract level using SEB smoothed rates indicated that within the high incidence risk counties presented in Figure 2, only a few census tracts displayed a high incidence risk of LACV infection (Figure 3). Thus, most of the census tracts in these seemingly high incidence risk counties actually had much lower incidence risks of LACV infection. Based on the smoothed rates it is clear that the geographic areas of highest incidence risk were clustered in a few census tracts in the western and northeastern parts of the study area. It is interesting to note that these spatial patterns seem to persist across all three population groups investigated (Figure 3).

### Geographic hot-spots of LACV infections

#### Global measures of clustering

The global Moran's I analysis did not identify any significant spatial clusters at the county level for all three population groups. However, evidence of significant (p<0.05) clustering was observed at the census tract level for each of the at-risk population groups. The computed global Moran's I values at the census tract level were 0.175 (p = 0.0004), 0.180 (p = 0.0008), and 0.186 (p = 0.0007) for the total population, the population 18 years and younger, and the population 15 years and younger, respectively.

#### Local measures of clustering

**Figure 4:**
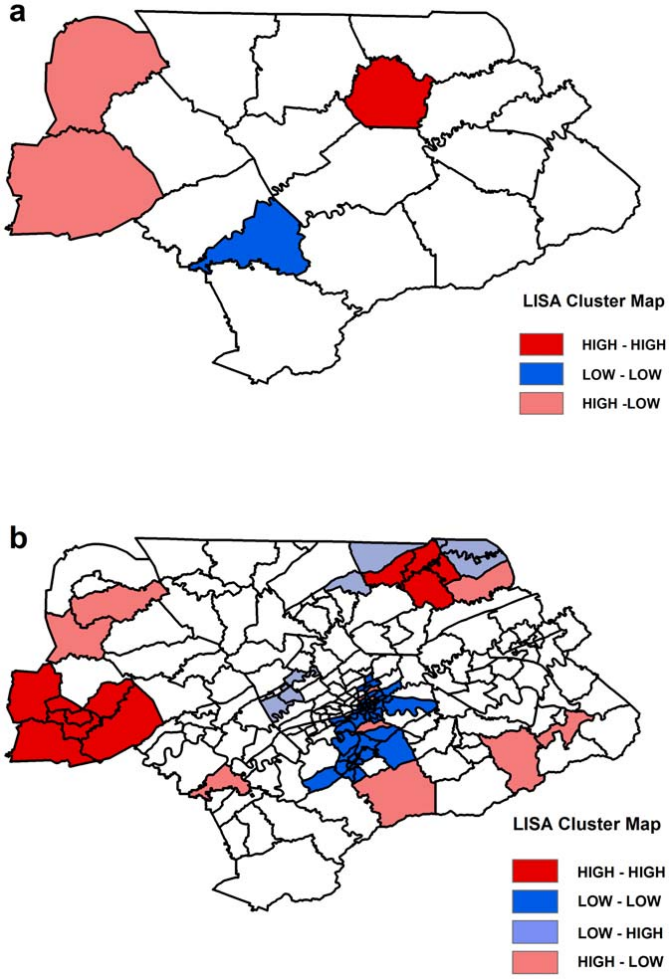
The spatial clustering of La Crosse virus infections at the county and census tract levels. These maps show the significant clustering of La Crosse virus infections for the population 18 years and younger at the county level (a) and at the census tract level (b) detected by the Local Indicators of Spatial Autocorrelation (LISA) statistic for eastern Tennessee.

Both positive and negative spatial autocorrelations were identified by LISA statistics. Positive spatial autocorrelation was considered to occur when high-risk counties/census tracts were surrounded by other high-risk counties/census tracts (“high-high”) or when low risk counties/census tracts were surrounded by other low risk counties/census tracts (“low-low”) (Figure 4). Negative spatial autocorrelation was considered to occur when high-risk counties/census tracts were surrounded by low risk counties/census tracts (“high-low”) or when low risk counties/census tracts were surrounded by high-risk counties/census tracts (“low-high”) (Figure 4).

Based on the above classification of positive and negative spatial autocorrelations, high-risk clusters (or hot-spots of LACV infections) were represented by “high-high” (Figure 4). Therefore, the LISA analysis at the county level identified a significant (p<0.05) local cluster in Union County for both the total population and the population 18 years and younger (Figure 4a). The results for the population 18 years and younger were similar to that of the total population, and therefore only the former has been presented in Figure 4. No significant (p>0.05) high-risk LACV infection local clusters were detected at the county level when using the population 15 years and younger as the at-risk population group. Significant (p<0.05) local clusters were identified at the census tract level using each of the three at-risk population groups (Figure 4b). Since all the at-risk populations yielded similar findings at the census tract level, only the results of the population 18 years and younger have been presented in Figure 4b. Twelve census tracts displayed evidence of significant (p<0.05) local clustering of high LACV infection risk for each of the three at-risk populations (Figure 4b). These significant high-risk clusters were located in Claiborne County (4 census tracts) and in Cumberland County (8 census tracts). The incidence risk of LACV infection in the 12 census tracts that displayed evidence of significant high risk spatial clustering were the highest among the population 15 years and younger and the lowest among the total population (Table 2).

**Table 2:** At-Risk Population Groups, Significant High Risk Clusters, and the Measures of Incidence Risk at the Census Tract Level for those Census Tracts Displaying Evidence of Spatial Clustering.

		Incidence Risk per 100,000 persons	
Population Groups	Significant High Risk Clusters*	Median	Range
Total population	12	33.2	15.2–133.3
Population 18 years and younger	12	157.5	59.4–547.1
Population 15 years and younger	12	177.6	68.0–673.9

* p<0.05.

## Discussion

This study highlights the variation that can occur when using different at-risk populations and geographic levels in the calculation of incidence risk and in the investigation of spatial patterns of disease distribution. These results further suggest a considerable increase in the incidence risk of LACV infection in eastern Tennessee and in those areas of highest risk. The incidence risks calculated in this study are the highest as of yet reported at the county level in Tennessee and at the census tract level nationally. The increased active surveillance activities initiated in 1997 may be responsible for decreasing underreporting and may partly explain the high LACV infection incidence risk in this study.

As anticipated the highest incidence risks were observed in the younger age groups. The population 18 years and younger and the population 15 years and younger both had considerably higher incidence risks when compared to the total population. The increased incidence risks within these populations highlights the importance of using not only crude and age-adjusted risks, but also age-specific risk calculations when investigating the epidemiology of LACV infections. The reason for the higher incidence risk in the pediatric population is not well understood, but could be due to differences in the pediatric and adult immune systems, viral dose, and/or exposure [38]. The estimates of the ratio of asymptomatic infections to symptomatic infections range from 2∶1 to 1500∶1 within the pediatric population in endemic areas [39], [40], [41]. As such, there are most likely several hundred thousand infections per year in the United States [39], [40], [41], although recent work suggests the number of cases may be higher [8].

The differences in LACV infection incidence risks between the county and census tract spatial levels were startling. The highest incidence risk in the population 15 years and younger at the county level was 226.4 per 100,000 persons and was as high as 673.9 per 100,000 persons at the census tract level. Although public health officials have traditionally reported the incidence risk of infection at the county level, the results of this study indicate that with focal diseases, such as LACV infections, analyses performed at a large geographic level may mask the underlying patterns of disease. Moreover, our findings indicate that the calculation and reporting of LACV infection incidence risk at these larger geographic levels (i.e. the county level) may lead to a distortion of the underlying spatial patterns of disease risk. This would be most apparent in focal diseases involving a small number of cases and affecting only a subset of the general population.

Statistically significant geographic hot-spots for all three populations were detected at the census tract level, even though this was not the case at the county level. It is clear that the observed spatial pattern of incidence risk will change according to the spatial level of analysis. Similar findings have been reported by other authors [42], [43], a phenomenon known as the modifiable areal unit problem (MAUP) [44]. The MAUP makes it advisable to perform the analyses at more than one spatial level, as has been done in this study. Had the analysis only been performed at the county level, as is usually the case, the observed spatial patterns would have been different from what was observed at the census tract level. It is therefore advisable to include the lowest possible geographic level in all analyses. However, when working with health data, there is often a need to aggregate the data to higher levels to protect patient privacy, though this must be done in a way so as not to mask existing spatial patterns in the data. Studies have shown that the use of aggregated data to investigate hot-spots of disease occurrence reduces the power to detect significant clusters [45]. Moreover, the more coarse the level of aggregation, the higher the reduction in power to detect true clusters [45].

The observed distribution of LACV infection hot-spots may reflect the spatial distribution of the primary disease vector, *Ae. triseriatus*, and secondary vector species, as well as the other risk factors that facilitate virus transmission. Some of these risk factors have been reported in other studies carried out in eastern Tennessee, and include the number of hours spent outdoors, the presence of mosquito larval habitats, standing water, tree-holes, vegetation, and the burden of vector species [23], [46]. Due to financial constraints, the current study did not investigate these risk factors. We have analyzed only those cases of clinical LACV infections that were reported to the health department. These cases are likely only a fraction of those exposed to infective vector species, and of those who developed clinical infections. Consequently, this distribution may not necessarily reflect the distribution of exposure and infection risks, which may be much higher.

Our findings both reaffirm and highlight the need for the use of the appropriate at-risk population and geographic levels of analysis, and the reporting of incidence risk when performing analyses on focal diseases that affect only a subset of the population. As such this study has four key findings: (a) using different at-risk populations aids in understanding the distribution of incidence risk of infection for LACV, as well as other diseases, (b) the geographic level of analysis affects the observed spatial patterns, (c) LACV infections cluster in certain localities in eastern Tennessee, and (d) the risk of LACV infections are considerably higher in eastern Tennessee than previously reported. Thus, the use of incidence risk maps of infection, spatial statistics, as well as the use of the most appropriate geographic level and at-risk population in the analyses of the incidence risk of infection will allow public health officials to better detect geographic hot-spots of infection and high risk population groups within these identified hot-spots. Employment of these strategies will enhance the targeting of limited resources to the highest risk groups resulting in the increased efficiency of surveillance, control, and prevention programs.

### Limitations

- We used the 2000 United States Census to provide the denominator for the computation of LACV infection incidence risk. This assumed that the age structure of the population was stable over the study period. However, if this assumption was not met, it could have influenced the computed incidence risks. It is our belief that even if the age structure was not stable, it would not have influenced the computed incidence risks significantly and the pattern of risk would not have changed accordingly.
- Probable and confirmed cases were reported through a passive surveillance system. Consequently, the incidence risks computed here are expected to be under-estimates of the true risk in the population. However, parents are more concerned about the health of their young children and are more likely to seek care when there is a complaint by the child. Thus, underreporting is not as prevalent in this age group resulting in a more accurate measure of incidence risk of infection. This underscores the importance of using the population 15 years and younger as the best at-risk group for computing LACV infection incidence risk.
- A downside to the use of the LISA in spatial analyses is the issue of multiple comparisons, which increase type I errors. These were not adjusted for because adjustments for type I errors result in increases in type II errors [31], [47]. Hence, such adjustments would lead to a reduction in the tests power to detect truly significant clusters [47].
